# New Occurrence of *Nigrospora oryzae* Causing Leaf Blight in *Ginkgo biloba* in China and Biocontrol Screening of Endophytic Bacteria

**DOI:** 10.3390/microorganisms12112125

**Published:** 2024-10-23

**Authors:** Yuan Tao, Chun Yang, Sinong Yu, Fangfang Fu, Tingting Dai

**Affiliations:** 1 Co-Innovation Center for the Sustainable Forestry in Southern China, Nanjing Forestry University, Nanjing 210037, China; 2 Modern Forestry Innovation Center of Yancheng State-Owned Forest Farm, Yancheng 224000, China

**Keywords:** plant disease, phylogenetic analysis, pathogenicity, endophytic biocontrol bacteria

## Abstract

*Ginkgo biloba* is a multifunctional composite tree species that has important ornamental, economic, medicinal, and scientific research value. In October 2023, the foliage of *G. biloba* on the campus of Nanjing Forestry University exhibited leaf blight. Black-brown necrotic spots were observed on a large number of leaves, with a disease incidence of 86%. After isolating a fungus from symptomatic leaves, pathogenicity was tested to satisfy Koch’s postulates. Using morphological features and multi-gene phylogenetic analyses of an internal transcribed spacer (ITS), elongation factor 1-alpha (*EF1-α*), and beta-tubulin (*β-tub*), the isolates YKB1-1 and YKB1-2 were identified as *Nigrospora oryzae. N. oryzae* was previously reported as an endophyte of *G. biloba*. However, this study shows it to be pathogenic to *G. biloba*, causing leaf spots. Two endophytic bacteria were isolated from asymptomatic leaves of diseased *G. biloba* trees, and their molecular identification was performed using 16S ribosomal DNA (16S rDNA). GBB1-2 was identified as *Bacillus altitudinis*, while GBB1-5 was identified as *Bacillus amyloliquefaciens*. The screening and verification of endophytic bacteria provide a new strategy for the control of *N. oryzae*.

## 1. Introduction

*Ginkgo biloba* L. is a deciduous tree of the genus *Ginkgo* in the family Ginkgoaceae, with an upright and graceful posture. It is an ornamental tree with high ornamental value, and due to its strong adaptability and high survival rate, it has been planted in large quantities on city streets in China [1,2]. *G. biloba* has important ornamental, economic, medicinal, and scientific value in China. It is one of the most widely planted street trees in China [3]. It can be used as a traditional Chinese medicine and also provides important value to scientific research fields such as botany and paleontology [4,5,6,7,8,9].

*Nigrospora* is a genus of fungal ascomycetes with a wide distribution and host range [10]. The genus name was first introduced by Zimmerman in 1902 for *Nigrospora panici*, an endophytic fungus isolated from the leaves of *Panicum amphibium* in Java, Indonesia [11]. It is now classified in the order Trichosphaeriales, class Pezizomycotina, phylum Ascomycota, kingdom Fungi. Currently, 51 species are described in www.mycobank.org. *Nigrospora* colonies spread quickly and are cotton-like and initially white, later turning brown, dark brown, or black. The conidiophores are conspicuous or inconspicuous, curved, colorless or light brown, and smooth. The conidial cells are single germinating, free, or solitary cells with limited growth. They are flask-shaped, colorless, and smooth. The conidia are terminal, simple, spherical or broadly ellipsoid, compressed, long, black, often shiny, and smooth, with the septa present or absent [12,13]. *Nigrospora* species are widely documented as plant pathogens of many economically important crops, fruits, and ornamentals. For example, leaf spot as well as black rot were observed in kiwifruit [14,15], leaf spot in olive [16], leaf blight in elephant grass [17], and reddish-brown spot disease in red-fleshed dragon fruit [18]. These diseases have been causing serious economic losses in agricultural production and forestry.

*Nigrospora* is also an endophytic fungus widespread in many animals and plants [19,20]. Fei et al. (2014) conducted a study on the biological characteristics of the rice spike rot pathogen in Anhui Province, China, reporting that the lethal temperature of one of the four pathogens, *Nigrospora oryzae*, was 59 °C, the optimal temperature for mycelial growth was 20–30 °C, and the optimal pH was 7 [21]. Li et al. (2016) concluded from the biological characterization of *N. oryzae* that it was able to grow at 20–35 °C and an average pH of 4.0–9.0, while its growth was significantly inhibited at 35 °C and pH 9.0 [22]. *N. oryzae* is a pathogenic fungus that can utilize a variety of carbon and nitrogen sources as food, is sensitive to alkaline environmental conditions, and is not resistant to high temperatures. Measures such as changing the habitat through the use of compost, the application of lime nitrogen disinfectants, and high-temperature greenhouses can effectively control the occurrence of the disease it causes.

The morphological characteristics of pathogenic fungi vary under different culture conditions, while the morphology of different fungal species can be highly similar. With the continuous development of molecular technology, identifying strains using a combination of morphological and molecular techniques has become a common strategy [23]. Based on the monophyletic relationship inferred from *LSU* sequence data (from the European large subunit ribosomal RNA database), Wang et al. (2017) found that *Nigrospora* is a monophyletic genus belonging to the Apiosporaceae. The authors then used a multi-locus genetic method based on ITS (internal transcribed spacers and 5.8S nuclear ribosomal DNA), *TEF1-α* (translation elongation factor 1-alpha), and *TUB2* (beta-tubulin), combined with morphological characters and ecological data, to classify and identify 165 Chinese isolates and 3 European isolates of *Nigrospora*, ultimately proposing a total of 13 new species, including 12 new species and one new combination [10]. Similarly, Wang et al. observed the morphology of the isolated rice leaf streak pathogen and identified it as *N. oryzae* by combining the ITS, *TEF1-α*, and *TUB2* gene sequences [24].

Previous studies have shown that there is a continuum of symbiosis between plants and endosymbiotic fungi, from mutualism to parasitism, which is influenced by many factors, including the transformation node, mode of infection, plant age, environmental conditions, and genetic background [25,26]. Endophytic fungi may become pathogenic during host senescence, although they are not considered pathogens in asymptomatic plants [27,28,29].

Several studies reported on the control of *N. oryzae*. The current control strategies for *N. oryzae* heavily rely on chemical interventions. However, the frequent use of chemical pesticides is associated with considerable environmental, health, and safety concerns [30,31]. Luo et al. (1987) used 40% kizazine, 40% Fuji-one, 40% edifenphos, 75% tricyclazole, 50% thiophanate methyl, and 50 μg/g of validamycin to control the rice round spot disease caused by *N. oryzae*; however, none of the above treatments achieved significant results [32]. Li et al. (2016) conducted a study on the mycelial growth and spore germination of *N. oryzae* in the presence of nine commonly used fungicides, determining that tetraconazole, trifloxystrobin combined with tebuconarole, and diphenoxazole combined with azoxystrobin were the most efficient [22]. However, the effect of their large-scale popularization and application in the field has yet to be further examined. Biological control refers to the use of beneficial organisms such as bacteria, fungi, and viruses to prevent and control diseases in forestry, agriculture, and other fields. This approach avoids polluting the environment and destroying the ecological balance [33]. The mechanisms of biological control include competition [34,35,36], antagonism [37], hyperparasitism [38], promotion of plant growth [39], induction of disease resistance [40], etc. Sempere et al. (2008) proved that *Penicillium oxalicum* can inhibit the growth of *N. oryzae* by penetrating and destroying its reproductive structure, making it deformed and unable to expand or reproduce [41]. Endophytic bacteria have co-evolved with plants for a long time and have become a natural component of the plant micro-ecosystem. They can promote the adaptation of plants to harsh environments and protect plants from infection by pathogens [42]. Yang et al. (2012) isolated *Bacillus amyloliquefaciens* from asymptomatic *G. biloba* leaves, revealing its ability to perform significant protection against pepper blight caused by *Phytophthora capsici* [43]. Thus, isolating endophytic bacteria with inhibitory effects against *N. oryzae* from asymptomatic leaves of *G. biloba* has become possible.

In October 2023, a undescribed *G. biloba* leaf disease was observed on the campus of Nanjing Forestry University (31°140′ N, 118°220′ E). Subsequently, the prevalence of this disease was immediately determined to be 86%. Black-brown necrotic spots accompanied by a yellow halo were observed in a large number of leaves. The diseased leaves eventually withered but did not fall off. Meanwhile, the infected plants exhibited stunted growth and a general reduction in overall vigor. Black fruiting bodies were observed at the wilting of the leaves, which seemed like conidiogenous cells of *Nigrospora*. The disease had a serious impact on both the ornamental and the economic value of *G. biloba*. The objectives of the current study were the following: (1) to isolate the pathogen; (2) to confirm its pathogenicity; (3) to determine the identity of the fungus; (4) to isolate and characterize endophytic bacteria antagonistic to the etiological agent.

## 2. Materials and Methods

### 2.1. Disease Investigation, Sampling, and Isolation

Twenty *G. biloba* leaves with leaf spots were collected from five symptomatic *G. biloba* trees on the campus of Nanjing Forestry University, China, in October 2023. The leaves were rinsed with sterile water for 25 min and then dried in sterile Petri dishes. Using sterile scissors, the leaves were cut into small pieces (3 mm × 3 mm) from the edge of the lesion, including both healthy and affected tissue. The pieces were treated in 75% ethanol for 30 s and in 1% NaClO for 90 s, rinsed five times in sterile water, dried on sterile filter paper, inoculated on potato dextrose agar (PDA), and incubated at 25 °C in the dark. After 3 days of incubation, the mycelium at the edge of the colony was cut and inoculated onto new PDA medium to obtain a pure culture.

### 2.2. Pathogenicity Tests

To test the pathogenicity of the isolates, fresh, healthy *G. biloba* leaves were collected in October, rinsed for 25 min with sterile water, and dried in sterile Petri dishes. The leaves were wounded in the distal area with a sterile needle (1 mm diameter, n = 10). Mycelial plugs of 6 mm diameter were cut from the edges of actively growing mycelium on fresh PDA and then set on the wound. The leaves were incubated in a dark incubator at 25 °C with moisture retention and examined 3, 5, 7, and 14 days post-inoculation (dpi). The pathogenicity test experiments were conducted three times.

Moreover, the pathogenicity of the isolates in the potted *G. biloba* seedlings was examined. Healthy leaves of one-year-old *G. biloba* seedlings (30 cm height, 2 wounds on each leaf; 6 leaves were tested) were wounded with a sterile needle (1 mm diameter) and inoculated with 6 mm plugs cut from the growing edges of 3-day-old cultures. The control plants were treated with PDA plugs. All inoculated leaves of the seedlings were covered with a polyethylene bag after the inoculation, and sterilized water was sprayed into the bags twice daily to maintain a humid microclimate. The temperature was maintained at 28 ± 2 °C. Three replicates were performed for each treatment and each control, and the pathogenicity experiments were performed in triplicate. The leaves used for the re-isolation were collected from the experimentally inoculated trees, and then the re-isolation of pathogens from both inoculated symptomatic leaves (one-year-old *G. biloba*, 25 pieces from 10 leaves) and uninoculated asymptomatic leaves (one-year-old *G. biloba*, 25 pieces from 10 leaves) was carried out according to the method described in Section 2.1; a comparison with the original pathogens was then performed following Koch’s postulates.

### 2.3. Morphological Identification

Plugs were obtained with a sterilized puncher of 6 mm diameter from the edge of pathogen colonies grown for three days and transferred to the center of dishes containing fresh PDA medium. The colonies were incubated in constant dark in an incubator at 25 °C for 5 d. The structure, color, and morphological characteristics of the colonies were observed and recorded.

To obtain a spore suspension, the conidia of the two isolates cultured on PDA medium for 20 days were washed off with sterile water. The morphology of the conidiophores and conidia of the two isolates were observed under an Axio Imager A2m microscope (Zeiss, Oberkochen, Germany). The size of the conidiophores and conidia of the two isolates was subsequently measured.

### 2.4. DNA Extraction, Amplification, Sequencing, and Phylogenetic Analyses

The fungal genomic DNA was extracted from the aerial mycelia of 5-day-old cultures using the cetyltrimethylammonium bromide protocol [44]. The extracted DNA was subjected to the polymerase chain reaction (PCR) amplification of partial regions of three genes/region, namely, the internal transcribed spacer (ITS) region, the elongation factor 1-alpha (*EF1-α*), and beta-tubulin (*β-tub*), which were amplified with the primers ITS1/ITS4 [45], EF1-728F/EF1-986R [46], and βt2a/βt2b [47], respectively. After the PCR, the products were sent to Shanghai Jieli Biotechnology Co. Ltd. (Shanghai, China) for DNA sequencing using the primers ITS1/ITS4, EF1-728F/EF1-986R, and βt2a/βt2b, respectively. Each region was sequenced five times. The PCR was conducted in 50 μL of the PCR mixture containing 19 μL of double-distilled water, 2 μL of genomic DNA (100 ng/μL), 2 μL of each primer (10 μmol/L), and 25 μL of Taq DNA polymerase mix (5 U/μL) (Takara Bio, Kyoto, Japan). The sequences of the two isolates (YKB1-1 and YKB1-2) were deposited in GenBank (Table 1). Table S1 reports the primers and PCR conditions used in this experiment.

The ITS, *EF1-α*, and *β-tub* sequences were compared to the sequences in GenBank using a BLAST search. The sequences of 31 *Nigrospora* isolates (14 species) were obtained from GenBank for phylogenetic analyses (Table 2), and the sequences of *Apiospora vietnamensis* (isolate ON426827.1) were used as an outgroup. The sequences of each gene/region were aligned using MAFFT ver. 7.313 [48] and manually adjusted through BioEdit ver. 7.0 [49]. The three genes/regions were combined in PhyloSuite ver. 1.2.2, while ModelFinder was used to select the best-fit model [50]. Phylogenetic relationships were inferred by maximum-likelihood (ML) analysis in IQtree ver. 1.6.8 with the bootstrapping method using 1,000,000 replicates [51]. Last, phylogenetic trees were drawn with FigTree ver. 1.4.4 (http://tree.bio.ed.ac.uk/software/figtree/, accessed on 10 July 2024).

### 2.5. Isolation of Endophytic Bacteria from G. biloba Leaves

Asymptomatic leaves from diseased *G. biloba* trees in the field were collected. Using sterilized scissors, the leaves were cut into small pieces (3 mm × 3 mm) and rinsed in sterile water for 25 min. Subsequently, the pieces were treated in 3% NaClO for 3 min, 75% ethanol for 5 min, and finally rinsed five times in sterile water for 2 min each time [52]. To check whether external sterilization was complete, the final wash was placed on LB medium and incubated for 48 h at 30 °C in an incubator. The cleaning was determined as complete if bacteria were absent from the sample. Following this, 10 tissue pieces were placed in a sterilized mortar with 1 mL of sterile water and ground thoroughly. The ground solution was evenly applied onto the surface of LB medium through the spread plate method. The medium was incubated for 48 h at 30 °C in the incubator to observe whether individual colonies grew on the medium. It was subsequently purified by the plate separation method [53], incubated for 48 h at 30 °C, and stored at 4 °C.

### 2.6. Screening for Endophytic Bacteria from G. biloba Leaves

A four-point plate standoff method was selected to screen for bacteria with antagonistic effects against *G. biloba* leaf blight pathogens [54]. Plugs were obtained with a sterilized puncher (6 mm diameter) from the edge of pathogen colonies grown for 3 days and transferred to the center of fresh PDA medium. Endophytic bacterial strains were inoculated at a distance of 1.5 cm from the plugs, with no endophytic bacterial strains used as a control. The medium was incubated at 28 °C for 4 d. The experiment was performed with three sets of replicates.

### 2.7. Molecular Identification of Endophytic Bacteria

The endophytic bacteria were inoculated in LB liquid medium and incubated at 160 rpm, 30 °C for 12 h. The endophytic bacteria were then incubated in LB liquid medium at 160 rpm, 30 °C for 12 h. Following this, 1 mL of the bacterial solution was placed into a centrifuge tube and centrifuged at 4 °C, 12,000 rpm/min for 5 min. The supernatant was discarded, and the solution was suspended in 100 μL of double-distilled H_2_O (ddH_2_O). The centrifuge tubes were then placed in a water bath at 100 °C for 5 min, which was immediately followed by a 10 min incubation at −20 °C. The water bath and freezing steps were then repeated. Last, centrifugation was performed at 4 °C, 12,000 rpm/min for 5 min to remove the precipitate, and the supernatant was the DNA of the endophytic bacteria.

The extracted DNA was subjected to PCR amplification of the bacterial 16S rDNA using the universal primers 27F and 1492R [55]. The PCR was conducted in 50 μL of PCR mixture containing 19 μL of double-distilled water; 2 μL genomic of DNA (100 ng/μL); 2 μL of each primer (10 μmol/L); and 25 μL of Taq DNA polymerase mix (5 U/μL) (Takara Bio, Kyoto, Japan). The 16S rDNA sequences of the two isolates (GBB1-2 and GBB1-5) were deposited in GenBank. Table S2 reports the primers and PCR conditions adopted in this experiment. After PCR amplification, the products were sent to Shanghai Jieli Biotechnology Co. Ltd. for DNA sequencing using the primers 27F and 1492R [55]. Each region was sequenced five times.

The sequences were compared to the sequences in GenBank using a BLAST search. The FASTA sequences of closely related species and isolates were downloaded from GenBank for phylogenetic analysis. The sequences of each region were aligned through ClustalW multiple alignment and then manually adjusted using BioEdit ver. 7.0.9.1 to ensure accuracy [49]. ModelFinder in PhyloSuite ver. 1.2.2 was used to select the best-fit model [50]. Phylogenetic relationships were inferred using ML analysis in IQtree ver. 1.6.8 under the AIC standard, employing bootstrapping with 1,000,000 replicates [51]. Phylogenetic trees were created with FigTree ver. 1.4.4 (http://tree.bio.ed.ac.uk/software/figtree/, accessed on 10 July 2024). Table 3 and Table 4 report the isolates and sequences used to identify GBB1-2 and GBB1-5, respectively.

## 3. Results

### 3.1. Symptoms Observed Under Field Conditions

**Figure 1:**
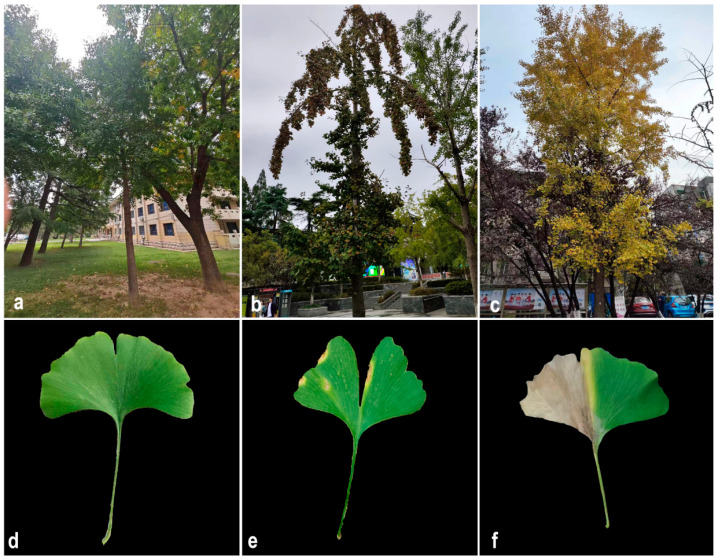
Symptoms observed in naturally infected *Ginkgo biloba* leaves. (**a**,**d**) Healthy *G. biloba* in the wild. (**b**,**e**) Early stage of *G. biloba* leaf blight in the wild. (**c**,**f**) Late stage of *G. biloba* leaf blight in the wild.

The prevalence of this disease was determined on campus to be 86%. The infected leaves initially formed a light brown spot, accompanied by a yellow halo on an indeterminate part of the leaves (Figure 1a,c). As the disease worsened, the area covered by the spots increased, and the spots gradually became black-brown from the center outward (Figure 1b,d). The diseased leaves eventually died but did not fall off. Moreover, after confirming the health of the symptomatic *G. biloba* roots, it was determined that symptomatic plants had stunted growth and a general decrease in overall vigor.

### 3.2. Pathogenicity of Fungal Isolates

**Figure 2:**
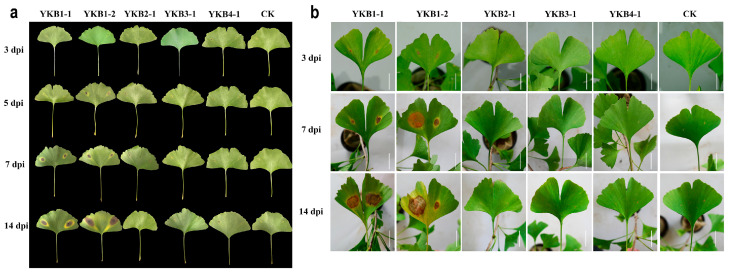
Pathogenicity of fungal isolates. (**a**) Morphology of inoculated *Ginkgo biloba* leaves in sterile Petri dishes 3, 5, 7, and 14 dpi. (**b**) Morphology of inoculated *G. biloba* leaves on one-year-old *G. biloba* seedlings 3, 7, and 14 dpi (scale bar = 2 cm).

A total of six isolates (YKB1-1, YKB1-2, YKB2-1, YKB3-1, and YKB4-1) were inoculated onto asymptomatic *G. biloba* leaves (Figure 2). Two isolates (YKB1-1 and YKB1-2) were confirmed as pathogenic to *G. biloba* leaves. Lesions appeared on wounds at 3 dpi. In addition, yellow longitudinal halos appeared around the lesions along the leaf veins. At 7 dpi, the diameter of the lesions caused by YKB1-1 increased to 1.2 ± 0.3 cm, while the diameter of the lesions caused by YKB1-2 increased to 1.8 ± 0.45 cm. Pycnidia were observed on the back of the lesions at 14 dpi. No lesions were observed on the leaves from the control plants. Meanwhile, the re-isolation results showed that the pathogens could only be isolated from diseased leaves and could not be isolated from non-diseased leaves of experimentally inoculated symptomatic plants.

### 3.3. Morphological Characteristics of the Pathogens

**Figure 3:**
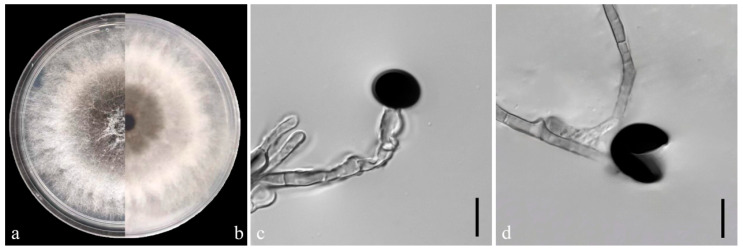
Morphological characteristics of YKB1-1 isolates. (**a**,**b**) Front and back views of a five-day-old colony of YKB1-1 on PDA. (**c**,**d**) Conidiogenous cells giving rise to conidia (scale bar = 10 μm).

**Figure 4:**
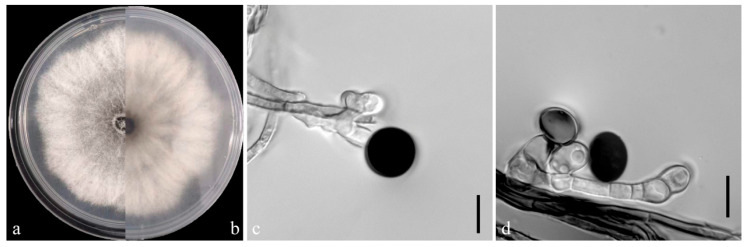
Morphological characteristics of YKB1-2 isolates. (**a**,**b**) Front and back views of a five-day-old colony of YKB1-2 on PDA. (**c**,**d**) Conidiogenous cells giving rise to conidia (scale bar = 10 μm).

On the PDA medium, the colonies of both pathogens (YKB1-1 and YKB1-2) were observed to be white in the top region. The hyphae were loose, extending radially. After 5 days, the colonies started to turn dark green from the center (Figure 3a,b and Figure 4a,b), and eventually the entire colonies turned dark green. The hyphae were observed to be branched, septate, transparent, and 2–6 μm in diameter (Figure 3c and Figure 4c). The conidia exhibited globose or subglobose shapes and were aseptate and solitary, forming on the top of transparent, bottle-shaped conidiogenous cells that later became black. The conidiogenous cells of YKB1-1 and YKB1-2 had dimensions of 4.2–12.8 μm × 3.1–7.2 μm (n = 50) and 3.7–11.7 μm × 3.3–6.6 μm (n = 50), respectively, and the corresponding conidia dimensions were 11.4–14.4 μm × 9.5–15.2 μm (n = 50) and 10.2–13.3 μm × 10.7–14.1 μm (n = 50), respectively.

### 3.4. Phylogenetic Analyses of the Pathogens

The genes/region ITS, *EF1-α*, and *β-tub* from the two isolates (YKB1-1, YKB1-2) were deposited into GenBank, and the accession numbers are presented in Table 1.

**Figure 5:**
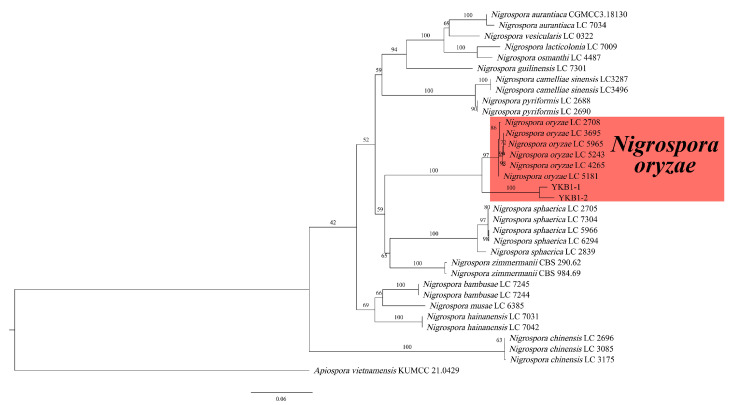
Phylogenetic relationship of YKB1-1 and YKB1-2 with related taxa derived from maximum-likelihood (ML) analysis using combined ITS, *EF1-α*, and *β-tub* sequence alignment of *Nigrospora* spp., with *Apiospora vietnamensis* (KUMCC 21.0429) as the outgroup. RA × ML bootstrap support values are shown at the nodes.

The genomic DNA of two pathogens isolates was amplified using the three primer pairs. The gel electrophoresis results showed that the size of the amplified genomic DNA was consistent with the expected size. The sequences were compared with the corresponding sequences in GenBank. The ITS sequence of YKB1-1 was 100% identical to that of the *N. oryzae* isolate C1W401C (GenBank accession no. MN783090), while that of YKB1-2 was 100% identical to that of the *N. oryzae* isolate 2-00367-3 (GenBank accession no. KT192361). The *EF1-α* sequence of YKB1-1 was 100% identical to that of the *N. oryzae* isolate LC7311 (GenBank accession no. KY019413), while that of YKB1-2 was 100% identical to that of the *N. oryzae* isolate LC7306 (GenBank accession no. KY019408). The *β-tub* sequence of YKB1-1 was 100% identical to that of the *N. oryzae* isolate xiao7h2-1 (GenBank accession no. MW562195), while that of YKB1-2 was 100% identical to that of the *N. oryzae* isolate LC5964 (GenBank accession no. KY019559). In the ML phylogenetic tree, the two isolates were in the same cluster with *N. oryzae* with 100% RA × ML bootstrap support values (Figure 5). Based on both multi-gene phylogeny and morphology, the two isolates (YKB1-2 and YKB1-5) were identified as *Nigrospora oryzae*.

### 3.5. Screening of Endophytic Bacteria for Biocontrol

**Figure 6:**
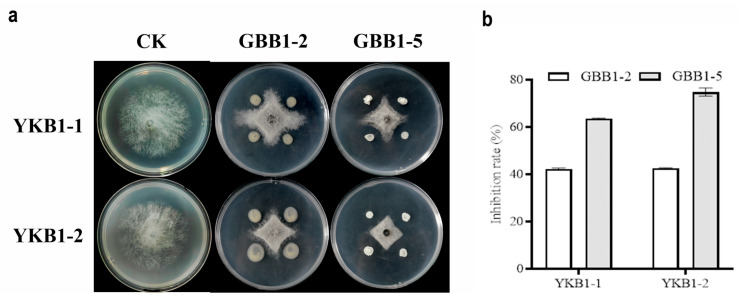
Screening results of endophytic bacteria for biological defense. (**a**) Inhibition of mycelial growth of *G. biloba* leaf blight pathogens by endophytic bacteria using the four-point plate standoff method. (**b**) Inhibition rate of pathogens by endophytic bacteria in the biological defense experiment.

Two strains with satisfactory inhibitory activity against YKB1-1 and YKB1-2 were isolated from asymptomatic leaves of diseased *G. biloba* trees. On PDA medium, both GBB1-2 and GBB1-5 significantly inhibited the hyphal growth of the two pathogens (Figure 6a). GBB1-2 inhibited YKB1-1 by 42.21%, whereas it inhibited YKB1-2 by 42.56%. GBB1-5 inhibited YKB1-1 by 63.55% and YKB1-2 by 74.79% (Figure 6b).

### 3.6. Phylogenetic Analyses of Endophytic Bacteria

**Figure 7:**
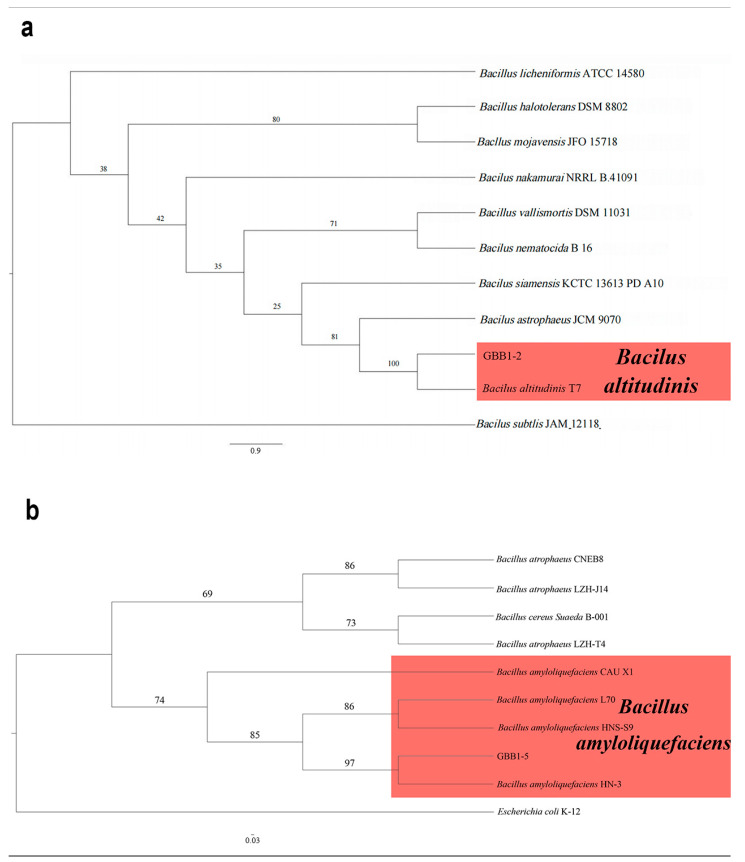
Phylogenetic relationship of selected endophytic bacteria with related taxa derived from maximum-likelihood (ML) analysis using 16s rRNA. (**a**) The phylogenetic relationship of GBB1-2. *Bacilus subtlis* (JAM 12118) was set up as the outgroup. RA × ML bootstrap support values are shown at the nodes. (**b**) The phylogenetic relationship of GBB1-5. *Escherichia coli* (K-12) was set up as the outgroup. RA × ML bootstrap support values are shown at the nodes.

The 16S rRNA gene sequences from the two endophytic bacteria (GBB1-2 and GBB1-5) were deposited into GenBank. The accession numbers are reported in Table 1. Following BLAST alignment analysis in NCBI, the sequence of GBB1-2 showed 100% identity to the corresponding one of *Bacillus altitudinis* (GenBank accession no. CP038517), while the sequence of GBB1-5 showed 100% identity to the corresponding one of *Bacillus amyloliquefaciens* (GenBank accession no. MW725248). As expected, in the ML phylogenetic tree, GBB1-2 clustered into the same clade as *B. altitudinis*, while GBB1-5 clustered into the same clade as *B. amyloliquefaciens* (Figure 7). Based on multi-gene phylogeny and morphology, GBB1-2 was identified as *B. altitudinis* (Figure 7a), and GBB1-5 was identified as *B. amyloliquefaciens* (Figure 7b).

## 4. Discussion

*G. biloba* not only is a widely planted ornamental foliage plant, but also possesses considerable medicinal, botanical, and paleontological value [4,5,6,7,8,9]. As the cultivated area of *G. biloba* increases, a greater number of diseases emerge. Stem rot in *G. biloba* due to *Maceophomina phaseoli* and *Trichothecium roseum* has been reported in China [56,57]. Previous studies identified *Alternaria tenuissima*, a *Colletotrichum* sp., a *Pestalotia* sp., *Botryosphaeria dothidea*, and *Dothiorella gregaria* as pathogens of *G. biloba* causing leaf blight [1,58]. However, there is a lack of research reporting *N. oryzae* as a cause of disease in *G. biloba*.

*Nigrospora* has been reported as an endophytic fungus of *G. biloba* [19,20]. Although not considered as pathogens in healthy plants, endophytic fungi can become pathogenic during host senescence [27,29]. In the current study, *N. oryzae* was identified as a pathogen of *G. biloba* causing leaf blight. Measurements by Wang et al. (2017) showed that the conidiogenous cells and conidia of *N. oryzae* have dimensions of 4–13 × 3–8.5 μm and 12.5–16 (mostly 12–14) μm, respectively [10], in agreement with the results of the isolates described here.

Previous research revealed that both *B. altitudinis* and *B. amyloliquefaciens* are endophytic bacteria of *G. biloba* [59,60]. Yu (2022) isolated *B. amyloliquefaciens* from *Hibiscus mutabilis*, achieving 67% inhibition of the mycelial growth of *N. oryzae* [61]. The isolated *B. amyloliquefaciens* also demonstrated a satisfactory inhibitory effect on the mycelial growth of *N. oryzae* in the current experiment. *B. altitudinis* possesses the capacity to impede the growth of a diverse array of fungal, bacterial, and oomycete organisms [60,62,63]. Nevertheless, the inhibitory effect of *B. altitudinis* on *N. oryzae* is yet to be investigated. The current study is the first to reveal the potential of *B. altitudinis* and *B. amyloliquefaciens* for the control of diseases caused by *N. oryzae*, providing a theoretical basis for the control of diseases in forest agriculture.

## 5. Conclusions

The current study classified pathogens causing leaf blight in *G. biloba.* Isolates were obtained from the infected leaves, and their pathogenicity was proved. The colony morphology, conidiogenous cells, and conidia of the isolates were observed. Furthermore, a phylogenetic tree was constructed based on the ITS, EF1-α, and β-tub multigene series. The pathogens were identified as *N. oryzae*. Two endophytic bacteria were isolated from asymptomatic leaves of infected *G. biloba* trees, and molecular identification was performed using 16S ribosomal DNA. GBB1-2 was identified as *B. altitudinis*, while GBB1-5 was identified as *B. amyloliquefaciens*, providing a strategy for the identification and prevention of this disease. In conclusion, the current study, on the one hand, firstly reports that *N. oryzae* causes leaf blight in *G. biloba*, widening the range of *G. biloba* leaf blight pathogens and providing a more comprehensive and systematic understanding of *G. biloba* leaf blight disease; on the other hand, it identified two endophytic bacteria of *G. biloba*, providing a basis for the prevention and control of *G. biloba* leaf blight.

## Figures and Tables

**Table 1:** Accession numbers of the isolates deposited in GenBank.

Isolate	DNA Target	GenBank Accession No.
YKB1-1	ITS	PQ047485
	*EF1-α*	PQ065936
	*β-tub*	PQ057798
YKB1-2	ITS	PQ047486
	*EF1-α*	PQ065937
	*β-tub*	PQ057799
GBB1-2	16s rDNA	PQ044816
GBB1-5	16s rDNA	PQ044817

**Table 2:** Isolates and sequences used to identify YKB1-1 and YKB1-2.

Species	Isolates	Host	Locality	GenBank Accession Number		
				ITS	*β-tub*	*EF1-α*
*N. aurantiaca*	CGMCC 3.18130 *	*Nelumbo* sp. (leaf)	China	KX986064	KY019465	KY019295
	LC 7034	*Musa paradisiaca*	China	KX986093	KY019598	KY019394
*N. bambusae*	LC 7244	Bamboo (leaf)	China	KY385306	KY385320	KY385314
	LC 7245	Bamboo (leaf)	China	KY385305	KY385321	KY385315
*N. camelliae-sinensis*	LC 3287	*Camellia sinensis*	China	KX985975	KY019502	KY019323
	LC 3496	*Camellia sinensis*	China	KX985985	KY019510	KY019327
*N. chinensis*	LC 2696	*Lindera aggregata*	China	KX985947	KY019474	KY019424
	LC 3085	*Camellia sinensis*	China	KX985970	KY019497	KY019427
	LC 3175	*Camellia sinensis*	China	KX985972	KY019499	KY019428
*N. hainanensis*	LC 7031	*Musa paradisiaca* (leaf)	China	KX986092	KY019597	KY019417
	LC 7042	*Musa paradisiaca* (leaf)	China	KX986094	KY019599	KY019418
*N. guilinensis*	LC 7301	*Nelumbo* sp. (stem)	China	KX986063	KY019608	KY019404
*N. lacticolonia*	LC 7009	*Musa paradisiaca* (leaf)	China	KX986087	KY019594	KY019454
*N. musae*	LC 6385	*Camellia sinensis*	China	KX986042	KY019567	KY019371
*N. oryzae*	LC 2708	*Rhododendron* sp.	China	KX985955	KY019482	KY019308
	LC 3695	*Osmanthus fragrans*	China	KX985988	KY019512	KY019329
	LC 4265	*Rhododendron* sp.	China	KX985994	KY019518	KY019335
	LC 5181	*Pentactina rupicola*	China	KX986032	KY019554	KY019359
	LC 5243	Submerged wood	China	KX986033	KY019555	KY019360
	LC 5965	Submerged wood	China	KX986038	KY019560	KY019364
*N. osmanthi*	LC 4487	*Hedera nepalensis*	China	KX986017	KY019540	KY019438
*N. pyriformis*	LC 2688	*Lindera aggregata*	China	KX985941	KY019468	KY019297
	LC 2690	*Rosa* sp.	China	KX985943	KY019470	KY019298
*N. sphaerica*	LC 7304	*Nelumbo* sp. (leaf)	China	KX986066	KY019610	KY019406
	LC 2705	*Rosa* sp.	China	KX985952	KY019479	KY019305
	LC 2839	*Harpullia longipetala*	China	KX985964	KY019491	KY019317
	LC 5966	Submerged wood	China	KX986039	KY019561	KY019365
	LC 6294	*Camellia sinensis*	China	KX986044	KY019565	KY019369
*N. vesicularis*	LC 0322	Unknown host plant	Thailand	KX985939	KY019467	KY019296
*N. zimmermanii*	CBS 290.62 *	*Saccharum officinarum* (leaf)	Ecuador	KY385309	KY385317	KY385311
	CBS 984.69	*Saccharum officinarum* (leaf)	Brazil	KY385310	KY385322	KY385316
*Apiospora vietnamensis*	KUMCC 21-0429	Bats	China	ON426827	OR025929	OR025968

*, ex-type cultures; ITS, internal transcribed spacers and intervening 5.8S nuclear ribosomal DNA; *β-tub*, beta-tubulin; *EF1-α*, translation elongation factor 1-alpha.

**Table 3:** Isolates and sequences used to identify GBB1-2.

Species	Isolate	GenBank Accession Number
*Bacillus altitudinis*	T7	OR285258
*Bacillus atrophaeus*	JCM 9070	NR024689
*Bacillus halotolerans*	DSM 8802	NR115063
*Bacillus licheniformis*	ATCC 14580	NR074923
*Bacillus mojavensis*	IFO 15718	NR024693
*Bacillus nakamurai*	NRRL B 41091	NR151897
*Bacillus nematocida*	B16	NR115325
*Bacillus siamensis*	KCTC 13613	NR 117274
*Bacillus subtilis*	IAM 12118	NR112116
*Bacillus vallismortis*	DSM 11031	NR024696

**Table 4:** Isolates and sequences used to identify GBB1-5.

Species	Isolate	GenBank Accession Number
*Bacillus amyloliquefaciens*	HN3	MK310270
	L70	OR841377
	CAU X1	OQ561751
	HNS S9	PP087014
*Bacillus atrophaeus*	CNEB8	MZ485377
	LZH-J14	MW474831
	LZH-T4	OL662992
*Bacillus cereus*	B-001	KT981877
*Escherichia coli*	K-12	LT899983

## Data Availability

The original contributions presented in the study are included in the article/Supplementary Materials, further inquiries can be directed to the corresponding author.

## Supplementary Materials

The following supporting information can be downloaded at: https://www.mdpi.com/article/10.3390/microorganisms12112125/s1, Table S1: Primer sequences and PCR amplification procedures used for identifying the pathogen; Table S2: Primer sequences and PCR amplification procedures used for identifying the endophytic bacteria.
